# Timing of the initiation of continuous renal replacement therapy and clinical outcome in patients with severe sepsis and septic shock

**DOI:** 10.1186/cc10970

**Published:** 2012-03-20

**Authors:** S Cho

**Affiliations:** 1 Seoul Asan Hospital, Seoul, South Korea

## Introduction

Timing of renal replacement therapy (RRT) in critically ill severe sepsis and septic shock patients with acute kidney injury is highly subjective and may influence outcome. The aim of this study is to evaluate the relationship between timing of RRT and 28-day mortality in patients with severe sepsis and septic shock.

## Methods

All patients diagnosed with severe sepsis and septic shock and treated at the medical ICU in a university-affiliated, tertiary-referral center, from January 2005 to December 2006 were reviewed. Timing of RRT was stratified into early and late by RIFLE (Risk, Injury, Failure, Loss, and End-stage) criteria and blood urea nitrogen (BUN) at the time RRT was started. The primary outcome was 28-day death from any cause.

## Results

Of the 326 patients diagnosed with severe sepsis and septic shock and admitted to the medical ICU during the study period, 78 patients received RRT. The mean age of the patients was 61.5 ± 14.7 years and 54 patients were male (69.2%). The timing of RRT was categorized into early (Risk, and Injury) and late (Failure) by RIFLE criteria and also categorized into early (BUN <75 mg/dl) and late (BUN ≥75 mg/dl). Comparing the relationship between RIFLE criteria (Risk and Injury vs. Failure) and 28-day mortality showed no significant difference (70.8% vs. 73.3%, *P *= 0.81). The timing of RRT by serum BUN also showed no significant difference in 28-day mortality before start of RRT by BUN ≥75 mg/dl versus BUN <75 mg/dl (77.3% vs. 69.6%, *P *= 0.50).

## Conclusion

Timing of RRT, stratified into early and late by RIFLE and BUN, showed no significant difference in 28-day mortality in patients with severe sepsis and septic shock.

